# The Neural Mechanisms Underlying the Decision to Rest in the Presence of Fatigue: A Magnetoencephalography Study

**DOI:** 10.1371/journal.pone.0109740

**Published:** 2014-10-10

**Authors:** Akira Ishii, Masaaki Tanaka, Yasuyoshi Watanabe

**Affiliations:** 1 Department of Physiology, Osaka City University Graduate School of Medicine, Osaka, Japan; 2 RIKEN, Center for Life Science Technologies, Hyogo, Japan; University of Michigan, United States of America

## Abstract

Adequate rest is essential to avoid fatigue and disruption of homeostasis. However, the neural mechanisms underlying the decision to rest are not well understood. In the present study, we aimed to clarify the neural mechanisms of this decision-making process using magnetoencephalography. Fifteen healthy volunteers participated in decision and control experiments performed in a cross-over fashion. In the decision experiment, participants performed 1,200 reverse Stroop test trials and were intermittently asked to decide whether they wanted to take a rest or continue. In the control experiments, participants performed 1,200 reverse Stroop test trials and were instructed to press a response button intermittently without making any decision. Changes in oscillatory brain activity were assessed using a narrow-band adaptive spatial filtering method. The levels of decrease in theta (4–8 Hz) band power in left Brodmann's area (BA) 31, alpha (8–13 Hz) band power in left BA 10 and BA 9, and beta (13–25 Hz) band power in right BA 46 and left BA 10 were greater in trials when the participant opted to rest (rest trials) than those in control trials. The decrease in theta band power in BA 31 in the rest trials was positively correlated with the subjective level of fatigue after the decision experiment. These results demonstrated that the dorsolateral prefrontal cortex, frontal pole, and posterior cingulate cortex play a role in the decision to rest in the presence of fatigue. These findings may help clarify the neural mechanisms underlying fatigue and fatigue-related problems.

## Introduction

Fatigue is a common problem in modern societies. Fatigue can be defined as difficulty initiating or sustaining voluntary activity [1]. More than 20–30% of the general population in European countries and the United States experience substantial fatigue [2]–[6], and in Japan, more than half of the adult population reports experiencing fatigue [7].

The unpleasant sensation that accompanies fatigue, i.e., fatigue sensation, plays an important role in biological alarm and urges us to take a rest to avoid disrupting homeostasis [8], [9]. Adequate rest is essential to avoid disrupting homeostasis. If individuals do not rest despite signs of fatigue they may experience overwork, which is a cause of death in a working population known as *karoshi* in Japan [10], [11]. Therefore, the decision of whether or not to rest based on the level of fatigue is important.

Although there have been several studies on the neural mechanisms of decision making in settings such as gambling (Bechara et al., 1994) and economic decision-making (Knutson et al., 2007; Smith et al., 2009), little is known about the neural mechanisms underlying the decision to rest in the presence of fatigue. The orbitofrontal cortex is one of the brain regions related to decision making [12], [13]. The regional cerebral blood flow in the medial orbitofrontal cortex, measured using H_2_^15^O positron emission tomography, was positively correlated with the level of fatigue sensation [8], indicating that this brain region may be involved in the decision to rest in the presence of fatigue. However, to the best of our knowledge, there have been no studies of the neural mechanisms underlying the decision to rest in the presence of fatigue to maintain task performance.

In the present study, we aimed to clarify the neural mechanisms underlying the decision to rest in the presence of fatigue. Participants performed a continuous cognitive task (i.e., reverse Stroop test trials) and were intermittently asked whether or not they wanted to take a rest in order to maintain their level of performance. Participants were instructed that they were free to take a rest whenever they thought that it was necessary. The neural activity present during the decision-making period was recorded with high temporal and spatial resolution using magnetoencephalography (MEG). We analyzed the MEG data using a narrow-band adaptive spatial filtering method [14], [15] and assessed the dynamics of oscillatory brain activities, which reflect time-locked cortical activities [16]–[18]. Since the neural mechanisms of the decision to rest in the presence of fatigue may involve multiple brain areas, high temporal resolution of the MEG and the information regarding the frequencies of oscillatory brain activities may provide important clues to clarify the role of each brain region involved in the neural mechanisms of decision to rest.

## Materials and Methods

### Participants

Fifteen healthy male volunteers (23.3±3.6 years of age [mean ± SD]) participated in this study. All participants were right-handed according to the Edinburgh Handedness Inventory [19]. Current smokers, individuals with a history of mental or brain disorder, and individuals taking chronic medications that affect the central nervous system were excluded. The Ethics Committee of Osaka City University approved the study protocol. All participants provided written informed consent for participation in this study in accordance with the principles of the Declaration of Helsinki.

### Experimental design

The study consisted of two experiments: a decision experiment and a control experiment. The two experiments were performed in a crossover fashion and on different days. The decision experiment consisted of reverse Stroop test trials and decision trials. The control experiment consisted of reverse Stroop test trials and control trials.

**Figure 1:**
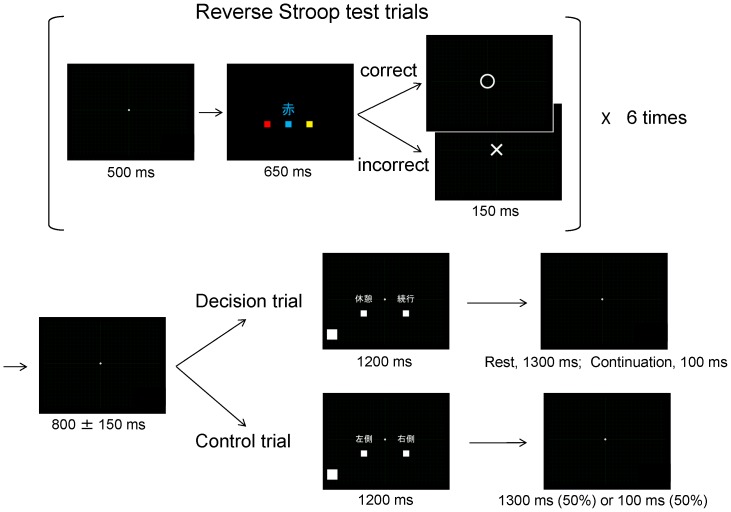
Experimental design. The study consisted of two experiments (decision experiment and control experiment) that were performed in a crossover fashion and on different days. The decision experiment consisted of reverse Stroop test trials and decision trials. The control experiment consisted of reverse Stroop test trials and control trials. The participants performed reverse Stroop trials and were intermittently asked to perform decision or control trials. An example of a reverse Stroop test trial is shown: In the example, the Japanese character corresponding to ‘red’ is presented in blue. In the decision experiment, a decision trial was performed once every six reverse Stroop test trials. In the decision trial, the participant was asked whether they needed a rest in order to maintain the performance of the reverse Stroop test. If they needed a rest they were instructed to press the left button of the response device with the index finger of their right hand (rest trial) and if they did not want a rest they were instructed to press the right button of the response device with the annular finger of their right hand (continuation trial). The buttons corresponding to the rest and continuation trials were indicated on the screen in Japanese. If the participant pressed the left button, the fixation dot was presented for 1,300 ms before the next reverse Stroop test trial started; otherwise, the fixation dot was presented for 100 ms before the next reverse Stroop test trial started. In the control experiment, a control trial was performed once every six reverse Stroop test trials. In the control trial, the participant was asked to press the left or right button with their index or annular finger, respectively. The correct button to press was indicated on the screen in Japanese. The duration of the fixation dot presented between the control trial and the next reverse Stroop test trials was 1,300 or 100 ms, determined at random. The mean ± SD interval between the reverse Stroop test trials and the decision and control trials was 800±150 ms: The jitter was generated based on Gaussian distribution. The total number of reverse Stroop test trials in each experiment was 1,200, and the number of decision or control trials was 200.

For both experiments, participants lay on a bed in a magnetically shielded room in the supine position, with a screen located in front of their eyes. Images were projected onto the screen by a video projector (PG-B10S; SHARP, Osaka, Japan). For reverse Stroop test trials, a fixation dot was presented on the screen for 500 ms, followed by one of three Japanese characters corresponding to ‘red’, ‘blue’, and ‘yellow’. The character was presented in red, blue, or yellow text (Figure 1). Participants were asked to indicate the meaning of the character, regardless of the color of the text. Responses were made by pressing the left, middle, or right button of a response device (HHSC-1×4-L; Current Designs, Philadelphia, PA) with the index, middle, or annular finger of the right hand, respectively. Participants were instructed to respond as quickly as possible and had a time limit of 650 ms in which to respond. Trials in which a button was not pressed within 650 ms were regarded as incorrectly answered. As soon as the participant pressed a button they were provided with feedback as to whether or not their response was correct (Figure 1). Feedback was presented for 150 ms, and was immediately followed by the fixation dot for the next trial. Participants performed 1,200 reverse Stroop test trials in each experiment.

In the decision experiment, a decision trial was presented once every six reverse Stroop test trials. In the decision trial, participants were asked if they needed a rest in order to maintain task performance. Participants were instructed that they were free to take a rest whenever they thought that it was necessary in order to maintain performance of the test trials. If they required a rest, they pressed the left button with their index finger (rest trials); otherwise they pressed the right button with their annular finger (continuation trials). For rest trials (left-button press), the fixation dot was presented for 1,300 ms before the next reverse Stroop test trial began. For continuation trials (right-button press), the fixation dot was presented for 100 ms before the next reverse Stroop test trial began. The total number of decision trials presented was 200.

In the control experiment, a control trial was presented once every six reverse Stroop test trials. In the control trial, participants were required to press either the left or the right button with their index or annular finger, respectively. The total number of control trials presented was 200. Just before the beginning of the control experiment, participants were instructed which button (left or right) they should press in response to control trials: Half the participants were instructed to press the right button in control trials 1–50, the left button in control trials 51–100, the right button control trials 101–150, and the left button in control trials 151–200, and the other half of the participants were instructed to press the button with the opposite pattern (left button in control trials 1–50 and 101–150, and right button in control trials 51–100 and 151–200). After a control trial, the fixation dot was presented for 1,300 or 100 ms before the next reverse Stroop test trial began, determined at random.

The mean ± SD interval between the reverse Stroop test trials and the decision and control trials was 800±150 ms. The jitter was generated based on a Gaussian distribution (Figure 1). Task trials used in the present study were created using OpenSesame software [20]. Just before and after each experiment, participants were asked to subjectively evaluate their level of fatigue on a visual analogue scale (VAS) ranging from 0 (minimum) to 100 (maximum). Just after each experiment, they were also asked to subjectively rate their level of fatigue they experienced during the experiment.

The experiments for eleven participants were performed between 3:00 p.m. and 6:00 p.m. and those for four participants were performed between 10:00 a.m. and 1:00 p.m.

### MEG recordings

MEG recordings were performed throughout the experiments using a 160-channel whole-head type MEG system (MEG vision; Yokogawa Electric Corporation, Tokyo, Japan) with a magnetic field resolution of 4 fT/Hz^1/2^ in the white-noise region. The sensor and reference coils were gradiometers with 15.5-mm diameter and 50-mm baseline, and the two coils were separated by 23 mm. The sampling rate was 1,000 Hz with a 0.3 Hz high-pass filter.

### MEG analyses

Before processing the MEG data, the magnetic noise that originated from outside the magnetically shielded room was eliminated by subtracting the data obtained from reference coils using specialized software (MEG 160; Yokogawa Electric Corporation). The MEG data corresponding to the rest, continuation, and control trials were separately averaged offline after analogue-to-digital conversion. Epochs of the raw MEG data that included artifacts were visually identified and were excluded from the analyses before averaging. Spatial filtering analysis of the MEG data was performed to identify the changes in oscillatory brain activity that reflected time-locked cortical activities [16]–[18] caused by performing the decision trials. The MEG data were bandpass filtered at 4–8 Hz, 8–13 Hz, 13–25 Hz, and 25–58 Hz by a finite impulse response filtering method using Brain Rhythmic Analysis for MEG software (BRAM; Yokogawa Electric Corporation) to obtain theta, alpha, beta and gamma signals, respectively. After the bandpass filtering, the location and intensity of the cortical activities were estimated using BRAM, which uses a narrow-band adaptive spatial filtering algorithm [14], [15]. Voxel size was set at 5.0×5.0×5.0 mm.

Oscillatory power ratios were calculated separately for the decision, rest, continuation, and control trials. The pre-stimulus time period (from 0 to 300 ms before the start of the decision, rest, continuation, and control trials) was used as the baseline. For each frequency band, oscillatory power was calculated from 227 to 27 ms prior to the onset of the button press (see Results for details) and was expressed relative to the oscillatory power in the baseline period (oscillatory power ratio). These data were then analyzed using statistical parametric mapping (SPM8, Wellcome Department of Cognitive Neurology, London, UK), implemented in Matlab (Mathworks, Sherbon, MA). The MEG parameters were transformed into the Montreal Neurological Institute T1-weighed image template [21] and applied to the MEG data. The anatomically normalized MEG data were filtered with a Gaussian kernel of 20 mm (full-width at half-maximum) in the x-, y-, and z-axes. To enable inferences to be made at a population level, individual data were summarized and incorporated into a random-effect model [22]. The weighted sum of the parameters estimated in the individual analysis consisted of “contrast” images, which were used for group analyses [22]. The resulting set of voxel values for each comparison constituted an statistical parametric map (SPM) of the t statistic (SPM{*t*}). The SPM{*t*} was transformed to the units of normal distribution (SPM{Z}). Significant signal changes from the control to the decision trials, and from the control to the rest and continuation trials, were assessed using *t* statistics on a voxel-by-voxel basis [22]. The threshold for the SPM{*t*} of group analyses was set at *P*<0.05 (corrected for multiple comparisons).

### Magnetic resonance (MR) image overlay

Anatomical MR imaging was performed using a Philips Achieva 3.0 TX (Royal Philips Electronics, Eindhoven, the Netherlands) to permit registration of magnetic source locations with their respective anatomical locations. Before MR scanning, five adhesive markers (Medtronic Surgical Navigation Technologies Inc., Broomfield, CO) were attached to the skin of the head: two markers 10 mm in front of the left tragus and right tragus, one marker 35 mm above the nasion, and two markers 40 mm either side of the marker above the nasion. The MEG data were superimposed on MR images using information obtained from these markers and MEG localization coils.

### Statistical analyses

Values are presented as mean and SD unless otherwise stated. Two-way analysis of variance (ANOVA) with repeated measures was performed to assess the effect of experiment (decision and control) and time period (before, during, and after the experiments) on the subjective level of fatigue. The relations among the change in power intensity of the MEG data from the control to the decision, rest, and continuation trials in Brodmann's area (BA) 31, BA 10, and BA 46 and the relation between the change in power intensity of the MEG data from the control to the decision, rest, and continuation trials in these brain regions and the subjective level of fatigue assessed just after the decision experiment were evaluated using Pearson's correlation. All *P* values were two-tailed, and values less than 0.05 were considered statistically significant. Statistical analyses were performed using IBM SPSS 21.0 software package (IBM, Armonk, NY).

## Results

### VAS scores

**Figure 2:**
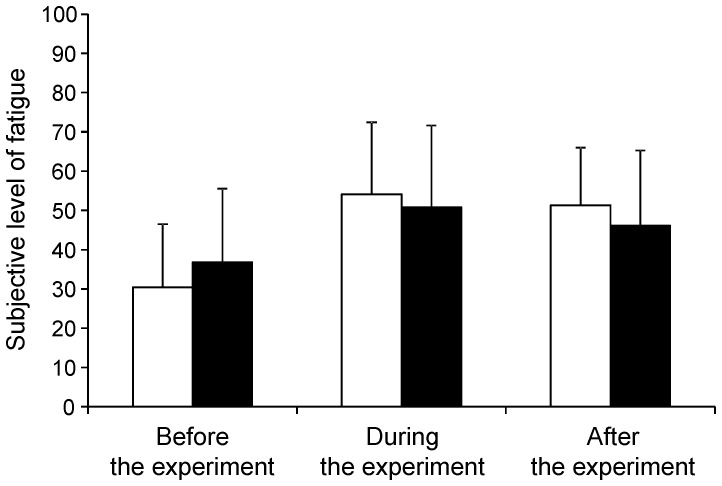
Subjective level of fatigue before, during, and after the decision and control experiments. Participants were asked to rate subjective level of fatigue before, during, and after the decision experiment (open columns) and the control experiment (closed columns) on a visual analogue scale from 0 (minimum) to 100 (maximum). Data are presented as mean and SD.

The subjective level of fatigue assessed using VAS before, during, and after decision and control experiments is shown in Figure 2. A two-way, repeated-measures AVNOVA was performed to assess changes in the subjective level of fatigue before, during and after the decision and control experiments. There was a main effect of time period [F(2, 28) = 6.744, *P* = 0.004] but no main effect of experiment [F(1, 14) = 10.678, *P* = 0.811]. There was a time course × experiment interaction [F(2, 28) = 4.191, *P* = 0.026].

### Spatial filtering analysis of MEG data

More than 6,000 time points are required to reliably estimate the peak intensities of the MEG data [15]. Trials with reaction time less than 200 ms were excluded from the analyses. The minimum reaction time among the analyzed rest, continuation, and control trials was 227 ms (subject No. 15 in the control trials). Subjects with less than 30 rest trials were also excluded from all analyses that included rest trials (n = 5; subject Nos. 1, 4, 5, 11, and 13). For the remaining trials, oscillatory power ratio was calculated for each frequency band for the time period of −227 to −27 ms (where zero is the onset of the button press). This ensured that all MEG data analyzed included more than 6,000 time points (200 ms×30 trials). The number of trials from each participant and the minimum, maximum, and mean reaction time after the rejection of the trials that included artifacts and after the exclusion of trials with reaction time less than 200 ms are shown in Table 1.

**Table 1:** Number of trials and minimum, maximum, and mean reaction time (RT) of the analyzed rest, continuation, and control trials.

SubjectNo.	Rest trials (Decision experiment)				Continuation trials (Decision experiment)				Control trials (Control experiment)			
	No.	Minimum RT (ms)	Maximum RT (ms)	Mean RT (ms)	No.	Minimum RT (ms)	Maximum RT(ms)	Mean RT (ms)	No.	Minimum RT (ms)	Maximum RT (ms)	Mean RT (ms)
1	8	-	-	-	129	264	974	439	138	239	803	429
2	50	617	1181	939	78	690	1196	933	59	691	1198	914
3	30	255	906	540	92	254	935	528	39	410	1064	628
4	12	-	-	-	139	253	818	417	165	255	573	407
5	14	-	-	-	102	236	685	381	130	255	863	445
6	37	412	1194	537	84	354	1194	524	125	255	1009	454
7	44	429	1008	655	111	444	994	604	168	487	1168	772
8	39	241	1168	700	96	241	1095	545	136	328	1199	666
9	37	245	1130	732	105	230	1173	581	149	280	1179	742
10	40	325	1107	661	102	281	1020	551	162	350	756	433
11	7	-	-	-	93	280	862	524	148	280	1020	515
12	58	309	859	571	114	294	961	546	155	400	1051	713
13	27	-	-	-	134	253	658	402	160	266	700	401
14	33	320	798	549	53	234	1162	584	95	400	1212	797
15	53	281	1005	616	113	252	1150	559	115	227	1081	622

Subject Nos. 1, 4, 5, 11, and 13 were excluded from the analyses of rest trials because the number of rest trials was <30.

To identify the brain regions associated with decision making, the oscillatory power ratio in theta, alpha, beta, and gamma frequency bands was compared between decision and control trials. The oscillatory power ratio in theta, alpha, beta and gamma frequency bands was similar in decision and control trials.

**Figure 3:**
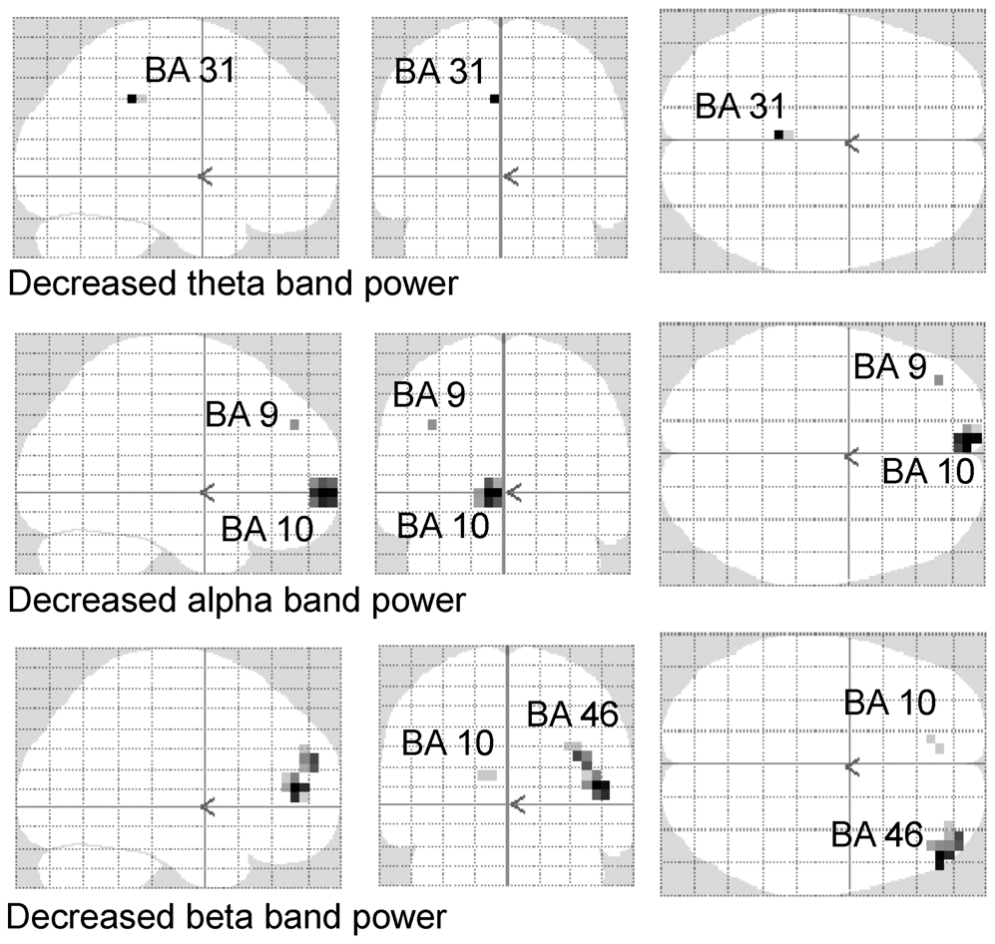
Maximum intensity projection of the statistical parametric maps. The level of decrease in theta (4–8 Hz), alpha (8–13 Hz), and beta (13–25 Hz) band power was greater in the rest trials than in the control trials. Random-effect analyses of 10 participants, *P*<0.05, corrected for the entire search volume (familywise error rate). BA, Brodmann's area.

To identify the brain regions associated with the decision to rest and the decision to continue, the oscillatory power ratio in theta, alpha, beta, and gamma frequency bands was compared between rest and control trials, and between continuation and control trials. Theta band power in left BA 31, alpha band power in left BA 10 and left BA 9, and beta band power in right BA 46 and left BA 10 was lower in rest trials than in control trials (Figure 3 and Table 2; *P*<0.05, corrected for multiple comparison using familywise error rate). The oscillatory power ratio in theta, alpha, beta and gamma frequency bands was similar in continuation and control trials.

**Table 2:** Brain regions that showed greater decrease in the oscillatory band power in the rest trials relative to the control trials.

Location	Frequency	Side	BA	MNI coordinates (mm)			Z value
				x	y	z	
Posterior cingulate gyrus	4–8 Hz	L	31	−3	37	40	4.13
Superior frontal gyrus	8–13 Hz	L	10	−8	63	0	4.31
Middle frontal gyrus	8–13 Hz	L	9	−38	48	35	4.12
Middle frontal gyrus	13–25 Hz	R	46	47	48	10	4.24
Medial frontal gyrus	13–25 Hz	L	10	−13	43	15	3.90

BA, Brodmann's area; MNI, Montreal Neurological Institute; L, left; R, right.

x, y, z: Stereotaxic coordinate.

Data were obtained from random-effect analyses. Only significant changes are shown (P<0.05, familywise error rate).

### Relation between subjective level of fatigue and decreases in power

**Figure 4:**
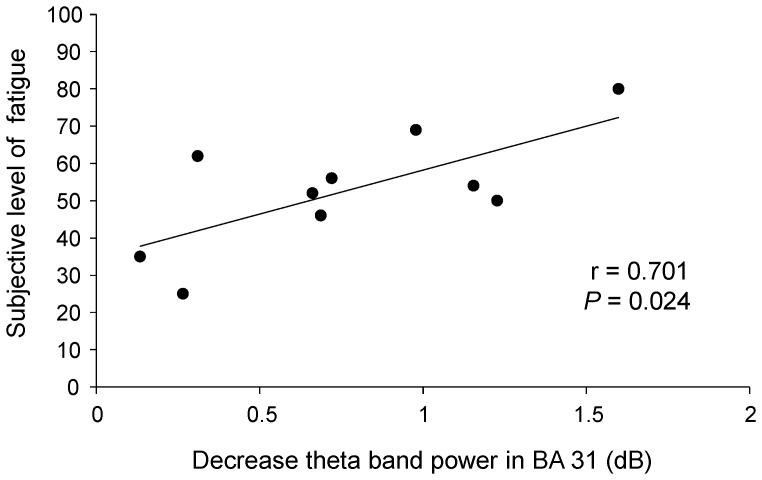
Relation between the decrease in theta band (4–8 Hz) power in Brodmann's area (BA) 31 in the rest trials and the subjective level of fatigue after the decision experiment. The linear regression line, Pearson's correlation coefficient, and *P* value are shown.

The decrease in the oscillatory power ratio in theta frequency band in BA 31 during the rest trials was positively correlated with the subjective level of fatigue just after the decision experiment (r = 0.701, *P* = 0.024; Figure 4).

### Relations among the decreases in power

**Figure 5:**
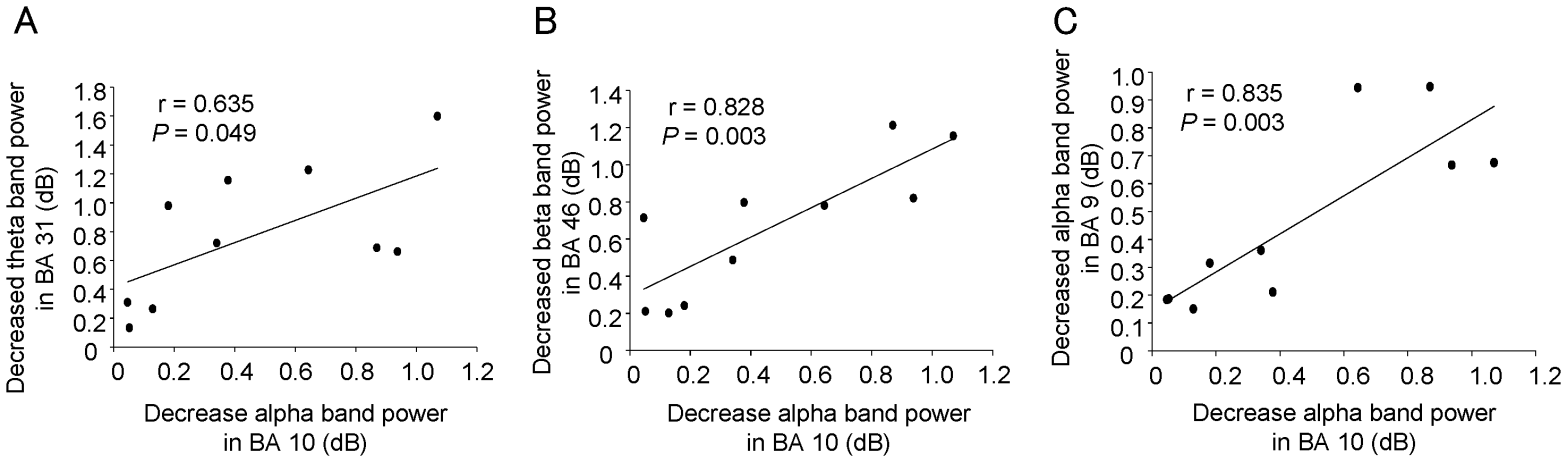
Relation between the decrease in alpha (8–13 Hz) band power in Brodmann's area (BA) 10 in the rest trials and the decrease in theta (4–8 Hz) band power in BA 31 (A) in the rest trials, the decrease in beta (13–25 Hz) band power in BA 46 (B) in the rest trials, and the decrease in alpha (8–13 Hz) band power in BA 9 (C) in the rest trials. Linear regression lines, Pearson's correlation coefficients, and *P* values are shown.

The decrease in the oscillatory power ratio in alpha frequency band in BA 10 during the rest trials was positively correlated with that in theta frequency band in BA 31 during the rest trials (r = 0.635, *P* = 0.049; Figure 5A), that in beta frequency band in BA 46 during the rest trials (r = 0.828, *P* = 0.003; Figure 5B), and that in alpha frequency band in BA 9 during the rest trials (r = 0.835, *P* = 0.003; Figure 5C).

## Discussion

In the present study, we showed that theta band power in BA 31, alpha band power in BA 10 and BA 9, and beta band power in BA 46 and BA 10 was decreased when participants made a decision to take a rest from a cognitive task. Power in these brain regions was not decreased when participants decided not to take a rest. The decrease in theta band power in BA 31 was positively associated with the subjective level of fatigue just after the decision experiment. In addition, the decrease in alpha band power in BA 10 was positively associated with the decrease in theta band power in BA 31 and the decrease in beta band power in BA 46.

The frontal pole (BA 10) is related to decision making and thinking of the future [23] and is involved in monitoring self-generated decisions [24]. The dorsolateral prefrontal cortex (DLPFC; BA 46 and BA 9) is related to decision making and response selection [25], [26], especially through response inhibition [27]–[29], and may also be involved in determining performance under conditions of fatigue [30]–[32]. The DLPFC is involved in the decision of whether to enhance or limit work output according to the situation. Decreases in alpha and beta band power are related to increases in neural activity [18], [33], therefore the decreased alpha and beta power observed in the frontal pole and DLPFC in the rest trials in the present study may indicate that activation of these brain regions was involved in the decision to take a rest under conditions of fatigue. This is supported by a report of decreased beta band power during decision making in a Go/No-go paradigm [29].

In addition to the frontal pole and DLPFC, we found that theta band power in the posterior cingulate cortex (PCC) was decreased when participants decided to take a rest, and this decrease was correlated with the subjective level of fatigue after the experiment. Several studies have shown that the PCC is related to the recognition of fatigue, i.e., fatigue sensation. Functional MR imaging studies have shown that the PCC and several other brain regions that were related to the subjective level of fatigue were activated in attention test trials [34], and the PCC was activated when participants imagined that they were fatigued [35]. Activation of the PCC was also observed when participants viewed pictures of people with fatigued expressions, suggesting involvement of the PCC in the neural mechanisms of fatigue recognition [36]. Recently, it has been suggested that the PCC is involved in self-evaluation of the level of fatigue, as the equivalent current dipoles in the PCC were related to self-evaluation of the level of physical fatigue and the equivalent current dipoles and decreased delta (1–4 Hz) band power in the PCC were related to self-evaluation of the level of mental fatigue in MEG studies [37], [38]. There have been several reports in which changes in delta and theta band power were observed in relation to emotional processing [39], [40]. Taking these facts into consideration, the role the PCC plays in the decision to rest or maintain task performance may be in the evaluation of the level of fatigue. According to the somatic marker hypothesis [41], [42], the PCC plays a critical role in decision making through triggering emotional responses [43]. Therefore, the important roles of the PCC for the decision to rest to maintain task performance based on the level of fatigue should be emphasized.

Changes in theta, alpha, and beta band power were observed only when the participants made the decision to rest, and not when the participants made the decision to continue without a rest. This can be explained as follows: The participants might not have hesitated to continue the task trials and might not have contemplated in the decision trials when they made decision to continue because they did not perform fatigue-inducing task trials, which can be reverse Stroop trials or other types of mental task trials such as 2-back task trials, prior to our experiments and thus their level of fatigue was low. On the other hand, making the decision to rest in the decision trials in our present study may have required more effort to appropriately estimate the level of fatigue, thus resulting in changes in theta, alpha, and beta band power. An alternative explanation why the activation of the DLPFC was observed only in the rest trials may be that the DLPFC is mainly related to the inhibitory processing in decision making [27]–[29].

Decreased theta band power in BA 31, decreased alpha and beta band power in BA 10, and decreased alpha band power in BA 9 were observed in the left side; decreased beta band power in BA 46 was observed in the right side. Our results were basically in line with previous studies: It has been reported that the left PCC and left BA 9 were related to the evaluation of the level of mental fatigue [44] and that the right dorsolateral prefrontal cortex was related to the response inhibition [27] and central inhibition during physical fatigue [30]. However, since decreased alpha and beta band power in the right BA 10 were observed when the statistical threshold of the SPM analyses was altered (P<0.001, uncorrected), we should be careful to refer to the laterality of the neural activities related to the neural mechanisms of the decision to rest.

We used the reverse Stroop test trials as the continuous cognitive task. Since the purpose of our present study was to clarify the neural mechanisms underlying the decision to rest in the presence of fatigue and only the neural activities during the decision-making period was analyzed, the reverse Stroop test trials used in our present study may be replaced by other kinds of mental task trials as long as the participants are able to make decisions to rest based on their level of fatigue.

There are limitations to our study. First, the number of the participants included in the analyses of the rest trials was limited. To generalize our results, studies with a larger number of participants are needed. Second, it is difficult to use MEG to reliably evaluate neural activity in brain regions that are located at the base of skull and outside the scope of the sensor helmet, such as the orbitofrontal cortex. Further studies with other neuroimaging modalities such as functional MR imaging are required to address activity in these brain regions. Third, as the participants did not perform fatigue-inducing task trials prior to the decision and control experiments, their level of fatigue was relatively low. It is of interest to determine whether neural activity related to the decision to take a rest differs when the subjective level of fatigue is high.

In conclusion, we showed that neural activity in the PCC, DLPFC, and frontal pole was related to the decision to take a rest. Our results suggest that, in addition to the prefrontal brain regions such as the DFPLC and frontal pole, the PCC also plays an important role in the decision to rest in the presence of fatigue. Our findings may help clarify the neural mechanisms underlying the decision to rest in the presence of fatigue and increase our understanding of the pathophysiology of fatigue and fatigue-related problems.
